# Utilization of breast cancer screening in Kenya: what are the determinants?

**DOI:** 10.1186/s12913-020-5073-2

**Published:** 2020-03-18

**Authors:** Roger Antabe, Moses Kansanga, Yujiro Sano, Emmanuel Kyeremeh, Yvonne Galaa

**Affiliations:** 1 Department of Geography, Western University, 1151 Richmond Street, Social Science Building, London, Ontario N6A 3K7 Canada; 2 Department of Sociology, Western University, 1151 Richmond Street, Social Science Building, London, Ontario N6A 3K7 Canada; 3 Department of Planning, Kwame Nkrumah University of Science and Technology, Private Mail Bag, University Post Office, KNUST, Kumasi, Ghana

**Keywords:** Breast cancer screening, Andersen’s behavioural model of health care utilization, Demographic and health survey, Kenya

## Abstract

**Background:**

Breast cancer accounts for 23% of all cancer cases among women in Kenya. Although breast cancer screening is important, we know little about the factors associated with women’s breast cancer screening utilization in Kenya. Using the Andersen’s behavioural model of health care utilization, we aim to address this void in the literature.

**Methods:**

We draw data on the Kenya Demographic and Health Survey and employ univariate, bivariate, and multivariate analyses.

**Results:**

We find that women’s geographic location, specifically, living in a rural area (OR = 0.89; *p* < 0.001) and the North Eastern Province is associated with lower odds of women being screened for breast cancer. Moreover, compared to the more educated, richer and insured, women who are less educated, poorer, and uninsured (OR = 0.74; p < 0.001) are less likely to have been screened for breast cancer.

**Conclusion:**

Based on these findings, we recommend place and group-specific education and interventions on increasing breast cancer screening in Kenya.

## Background

In many sub-Saharan African (SSA) countries, breast cancer remains the leading cause of cancer-related morbidity and mortality among women. According to the 2015 Kenya Stepwise Survey of Non-Communicable Disease Risk Factors Report, the situation is not different in Kenya, as breast cancer is the most common cancer (34 per 100,000), accounting for 23% of all cancers of women [1]. In this context, early screening for breast cancer has been recommended as a strategy to mitigate the associated morbidity and mortality. Although the World Health Organization (WHO) recommends mammography as the most effective way for the early diagnoses and subsequent treatment of breast cancer, in low income setting where health care systems may be weak, it observes that clinical examination is a promising approach given its cost effectiveness [2, 3]. In Kenya, clinical breast examination is recommended by policy as it offers an opportunity to educate women about breast health although it is not to be considered a replacement for mammography [4]. Thus, in low income settings of SSA, through early screening programs, which often include clinical examination, health professionals can understand women’s health status, risk levels, and susceptibility to breast cancer for appropriate intervention strategies such as early treatment and counselling [5]. Accordingly, the Kenyan government established the National Cancer Control Strategy, a set of interventions aimed at providing a comprehensive national cancer screening program, improved access to medicines and building a population-based cancer registry.

While the national-level program advocates for education and mass screening activities at the community level, breast cancer screening rate for women has unfortunately remained low in Kenya [4, 6]. For instance, it is reported that 86% of women in Western Kenya did not undergo any breast cancer screening previously [6]. Furthermore, Akinyemiju [4] found that the screening rate for breast cancer is about 5% in Kenya making it difficult for early detection among majority of Kenyan women. Considering the low usage rate, it is critical to understand factors associated with women’s uptake of breast cancer screening at the national level. However, Naanyu et al. [5] suggest that population-based data on breast cancer and breast cancer screening have been largely unavailable in many developing countries. The 2014 Kenya Demographic and Health Survey which is a national-level survey provides a unique opportunity to explore this topic in the context of developing countries such as Kenya. To this end, the current study aims to advance the literature by exploring demographic and socioeconomic correlates of women’s breast cancer screening utilization in Kenya.

We use the Andersen’s behavioural model of health care utilization as a conceptual framework [7]. According to this framework, there are three clusters of factors that may promote or hinder people’s utilization of health care. The first component includes predisposing characteristics, which point to the important role of social structure, health beliefs, and social support on healthcare utilization. The second component emphasizes enabling characteristics, which include availability and financial means to access healthcare services. The last component includes need characteristics, suggesting that people are more likely to use healthcare services when they perceive the need to seek medical treatment for a given health condition. In the following sections, we explore how these three clusters of factors may contribute to women’s breast cancer screening utilization in Kenya.

### Predisposing characteristics

A number of predisposing characteristics may influence women’s breast cancer screening utilization. For example, in many African countries including Kenya, educational attainment is considered an important predictor of women’s use of formal and preventive health care services including cancer screening, given its role in knowledge provision on health and its related behaviours [8, 9]. Reflecting on the relatively low levels of education among women in developing countries, there may be a significant knowledge gap on cancer and cancer screening in Kenya. Specifically, Muthoni and Miller [7] have emphasised the importance of delivering educational programs on breast cancer in community settings, as women with low level of education often lack knowledge and awareness about breast cancer and breast cancer screening. Thus, it is possible that there are educational disparities in breast cancer screening utilization among women in Kenya.

Moreover, marital status may be important for women’s uptake of breast cancer screening. Marriage can promote healthy behaviours for at least two reasons. First, married people’s health behaviours are often monitored and shaped by their spouses [10]. Second, marriage can provide emotional support for maintaining healthy behaviours [11]. Consequently, research suggests that married people are more likely to adapt preventive behaviours including eating balanced meals, engaging in regular exercise, and utilizing preventive healthcare services including breast cancer screening [12]. In Kenya, married women are more likely to attend antenatal care and to have more antenatal care visits compared to unmarried women [13]. Given the linkage between formal and preventive healthcare utilization and marital status, unmarried women may be less likely to use breast cancer screening than married women.

In addition, due to the complex relationships linking religion, ethnicity, and location, the province of residence may influence women’s uptake of breast cancer screening. For instance, religious norms and practices may discourage Muslim women from undergoing breast cancer screening, particularly from male health practitioners [14]. This may be the case in the North Eastern Province where the population is predominantly Muslim and Somali [15]. Moreover, Somali people are largely nomadic pastoralists, being physically distant from the dominant sedentary society and occupying peripheral areas where health facilities may be lacking [16]. In addition to these religious and ethnic characteristics, Kumssa, Jones and Williams [17] emphasize that the North Eastern Province is one of the most underdeveloped regions in Kenya, with more than three-quarters of the population living below the poverty line. Considering their religious, ethnic, and economic standings, we expect that women in the North Eastern Province are less likely to have been examined for breast cancer than women in other provinces.

Also, in Kenya, Lesotho, and Swaziland research shows that lack of medical infrastructure and services such as screening programs and cancer treatment facilities coupled with inadequate health professionals hinder women’s uptake of cancer screening services [18]. Research suggests that, in most developing countries where resource provision including health facilities are skewed in favour of urban areas [19, 20], women in rural areas may have limited opportunities to be screened for breast cancer compared to those in urban areas. Moreover, increased distance from health service providers often serves as a barrier for healthcare utilization [21, 22]. The problem of proximity or distance may be reinforced by the poor transportation networks linking rural areas to health facilities in many developing countries including Kenya.

### Enabling characteristics

Financing cancer screening may be difficult in Kenya, as research shows that a significant proportion of women reported financial barriers to cancer screening in a health facility [23, 24]. Although Kenya’s national health insurance covers a wide range of cost for health care services in governmental hospitals, only a small proportion of Kenyans (7%) are enrolled [25]. This may be due to the non-consolidation of multiple health insurance schemes that may not be socially oriented. Furthermore, these schemes may be prioritizing the enrolment of formal sector workers to the neglect of the informal sector which is the largest employer [25, 26]. Thus, it may not be reaching the majority of women, who may require financial aid to undergo breast cancer screening [27]. To this end, we expect that poorer and uninsured women are less likely to have been screened for breast cancer than their richer and insured counterparts.

### Need characteristics

It is also documented that people’s healthcare seeking behaviours including cancer screening may be influenced by perceptions of health risk [28]. More specifically, some women may not consider themselves at risk and therefore do not recognize the importance to undergo breast cancer screening. Furthermore, it is known that age is an important biological determinant of breast cancer as evidence suggest that the peak age for breast cancer in Kenya is 50–59 years old [1]. Given the age-specific nature of breast cancer prevalence in Kenya, it is possible that older women may perceive the risk of breast cancer more than younger women. Consequently, younger women may be less likely to use breast cancer screening than older women.

## Methods

We drew on data from the 2014 Kenya Demographic and Health Survey (KDHS), a nationally representative survey of women aged 15 to 49 and men aged 15 to 54. It was implemented by the Kenya National Bureau of Statistics (KNBS) in collaboration with the Ministry of Health, the National AIDS Control Council (NACC), the National Council for Population and Development (NCPD), and the Kenya Medical Research Institute (KEMRI), with technical assistance from ICF International. The KDHS provides reliable demographic and health indicators including women’s access to breast cancer screening. The KDHS employed a two-stage sampling design in which a stratified probability proportional to size sampling methodology was applied. Face-to-face interviews were conducted with 14,741 women and 12,819 men with a response rate of 96 and 90%, respectively. For the purpose of this study, we focused on 14,734 women who answered questions on their access to breast cancer screening.

### Measures

Respondents were asked whether they ever had their breast examined to detect or check for breast cancer by a health professional. We used this question as the dependent variable (0 = no; 1 = yes). Following the Andersen’s behavioural model of health care utilization, we introduced three sets of explanatory variables, namely predisposing, enabling, and need factors. For predisposing factors, we included level of education (0 = higher education; 1 = secondary education; 2 = primary education; 3 = no education), marital status (0 = currently married; 1 = never married; 2 = formerly married), province of residence (0 = North Eastern; 1 = Coast; 2 = Eastern; 4 = Rift Valley; 5 = Western; 6 = Nyanza; 7 = Nairobi), place of residence (0 = urban; 1 = rural), and employment status (0 = employed; 1 = unemployed). There were two enabling factors, which include household wealth quintiles (0 = richest; 1 = richer; 2 = middle; 3 = poorer; 4 = poorest) and insurance status (0 = insured; 1 = uninsured). Household wealth quintiles were created from a composite index based on a number of household ownership items including drinking water, car ownership, and toilet facilities, among others. Finally, we included one need factor, which is age of respondents (0 = 45–49; 1 = 40–44; 2 = 35–39; 3 = 30–34; 4 = 25–29; 5 = 20–24; 6 = 15–19).

### Data analysis

We employed univariate, bivariate, and multivariate analyses. Although our dependent variables were dichotomous, as shown in Table 1, higher categories (1 = yes) were much less probable than lower categories (0 = no). In this case, using a simple logit link function, which assumes symmetry, could potentially produce biased parameter estimates [29]. Thus, for bivariate and multivariate analyses, we used the negative log-log model that is considered suitable when the higher category of the dependent variable is less probable than the lower category. Moreover, due to the multistage sample design, complex population-based surveys such as the KDHS usually have some degree of dependence among the observations. This can be problematic because standard regression models such as ours often assume independence among the observations [30]. Using the ‘vce’ option provided by STATA, we accounted for this potential bias by imposing a ‘cluster’ variable, which constitutes the unique identification numbers assigned to respondents at the cluster level. Findings were reported in odds ratios (ORs). ORs larger than 1 indicate higher odds of having been screened for breast cancer, while those smaller than 1 indicate lower odds of having done so.

**Table 1:** Univariate analysis of dependent and independent variables

	Counts	Percentage
**Ever screened for breast cancer**		
No	13,037	88
Yes	1697	12
**Level of education**		
Higher education	1251	8
Secondary education	4104	28
Primary education	7399	50
No education	1980	14
**Marital status**		
Currently married	9010	61
Never married	4056	28
Formerly married	1668	11
**Province of residence**		
North Eastern	779	5
Coast	1841	13
Eastern	2494	17
Central	1511	10
Rift Valley	4251	29
Western	1385	9
Nyanza	2013	14
Nairobi	460	3
**Urban-rural residence**		
Urban	5469	37
Rural	9265	63
**Employment status**		
Employed	8447	57
Unemployed	6270	43
**Household wealth quintiles**		
Richest	2946	20
Richer	2947	20
Middle	2947	20
Poorer	2947	20
Poorest	2947	20
**Insurance status**		
Insured	2240	15
Uninsured	12,489	85
**Age of respondents**		
45–49	1127	8
40–44	1370	9
35–39	1876	13
30–34	2105	14
25–29	2858	19
20–24	2537	17
15–19	2861	20
Total	14,734	100

## Results

### Univariate analysis

Table 1 shows findings from univariate analysis. We found that 12% of Kenyan women have been screened for breast cancer. Although 57% were employed, very few women attained higher education (8%). Similarly, nearly one fourth of women (24%) were from the poorest households and the majority did not have any health insurance (85%). Three fifth of women were currently married (61%), while the largest age group was between 15 to 19 years old (20%). Also, more women lived in rural areas (63%) than urban areas (37%), and the largest proportion of women lived in the Rift Valley Province (29%), followed by Eastern (17%), Nyanza (14%), and Coast (13%) Province.

### Bivariate analysis

Table 2 shows findings from bivariate analysis. We found predisposing, enabling, and need factors are largely significantly associated with breast cancer screening. For example, women with lower levels of education were less likely to have been screened for breast cancer than their counterparts with higher levels of education. Never married women were also less likely to have been screened for breast cancer than currently married women (OR = 0.74, *p* < 0.001). Moreover, women residing in the North Eastern Province were least likely to have been screened for breast cancer screening than women in any other province. In addition, women in rural areas were less likely to have been screened for breast cancer than those in urban areas (OR = 0.76, *p* < 0.001). Unemployed women were also less likely to have screened for breast cancer than employed women (OR = 0.69, p < 0.001). Furthermore, poorer and uninsured were less likely to have been screened for breast cancer than their richer and insured counterparts. Finally, women aged 15–19 (OR = 0.55, p < 0.001) and 20–24 (OR = 0.83, p < 0.001) were less likely to have been screened for breast cancer than those aged 45–49.

**Table 2:** Bivariate analysis of dependent and independent variables

	OR (95% CIs)
**Level of education**	
Higher education	1.00
Secondary education	0.61 (0.56, 0.66)***
Primary education	0.55 (0.51, 0.59)***
No education	0.34 (0.31, 0.38)***
**Marital status**	
Currently married	1.00
Never married	0.74 (0.70, 0.78)***
Formerly married	1.01 (0.95, 1.08)
**Province of residence**	
North Eastern	1.00
Coast	1.65 (1.42, 1.91)***
Eastern	1.86 (1.61, 2.14)***
Central	3.24 (2.79, 3.75)***
Rift Valley	1.91 (1.66, 2.20)***
Western	1.75 (1.50, 2.03)***
Nyanza	1.65 (1.42, 1.91)***
Nairobi	2.63 (2.20, 3.13)***
**Urban-rural residence**	
Urban	1.00
Rural	0.76 (0.73, 0.79)***
**Employment status**	
Employed	1.00
Unemployed	0.69 (0.66, 0.72)***
**Household wealth quintiles**	
Richest	1.00
Richer	0.81 (0.76, 0.86)***
Middle	0.69 (0.65, 0.74)***
Poorer	0.62 (0.58, 0.66)***
Poorest	0.43 (0.41, 0.48)***
**Insurance status**	
Insured	1.00
Uninsured	0.55 (0.52, 0.58)***
**Age of respondents**	
45–49	1.00
40–44	1.03 (0.93, 1.13)
35–39	0.99 (0.90, 1.08)
30–34	1.00 (0.92, 1.10)
25–29	0.98 (0.90, 1.07)
20–24	0.83 (0.76, 0.91)***
15–19	0.55 (0.50, 0.60)***

***p < 0.001; OR for odds ratio; 95% CIs = 95% confidence intervals

### Multivariate analysis

Although bivariate findings are useful, they are limited to the gross impacts of the independent variables on the dependent variable. We further employed multivariate analysis to estimate the net impacts. Findings from multivariate analysis are shown in Table 3. Consistent with bivariate findings, multivariate analysis shows that predisposing, enabling, and need factors were largely significantly associated with breast cancer screening. In Model 1, we found that women with lower levels of education were less likely to be screened for breast cancer than those with higher levels of education. Also, never married women were less likely to have been screened for breast cancer than currently married women (OR = 0.68, *p* < 0.001). Moreover, women from North Eastern Province were least likely to have been screened for breast cancer than those from any other province. We also found that unemployed (OR = 0.85, *p* < 0.001) and rural women (OR = 0.83, p < 0.001) were less likely to have been screened for breast cancer than their employed and urban counterparts. In Model 2, we further controlled for enabling factors. We found that poorer women were less likely to have been screened for breast cancer than richer women. Similarly, uninsured women were less likely to have been screened for breast cancer than insured women (OR = 0.73, *p* < 0.001). It is noteworthy that the impact of employment status on breast cancer screening was largely attenuated once the enabling factors were controlled in Model 2 (OR = 0.93, *p* < 0.05). Finally, we also found in Model 3 that women aged 15–19 (OR = 0.74, p < 0.001) were less likely to have been screened for breast cancer than those aged 45–49.

**Table 3:** Multivariate analysis of ‘ever tested for breast cancer’ among women in Kenya

	Model 1	Model 2	Model 3
	OR (95% CIs)	OR (95% CIs)	OR (95% CIs)
**Level of education**			
Higher education	1.00	1.00	1.00
Secondary education	0.64 (0.59, 0.70)***	0.73 (0.67, 0.80)***	0.77 (0.70, 0.84)***
Primary education	0.56 (0.51, 0.60)***	0.69 (0.63, 0.76)***	0.72 (0.65, 0.78)***
No education	0.39 (0.34, 0.43)***	0.55 (0.48, 0.62)***	0.55 (0.48, 0.62)***
**Marital status**			
Currently married	1.00	1.00	1.00
Never married	0.68 (0.64, 0.72)***	0.70 (0.66, 0.74)***	0.80 (0.74, 0.86)***
Formerly married	0.99 (0.93, 1.07)	1.03 (0.96, 1.10)	1.02 (0.95, 1.10)
**Province of residence**			
North East	1.00	1.00	1.00
Coast	1.34 (1.13, 1.60)***	1.40 (1.17, 1.68)***	1.40 (1.16, 1.68)***
Eastern	1.47 (1.24, 1.75)***	1.48 (1.24, 1.78)***	1.49 (1.24, 1.79)***
Central	2.40 (2.01, 2.87)***	2.33 (1.94, 2.80)***	2.34 (1.94, 2.82)***
Rift Valley	1.52 (1.28, 1.80)***	1.55 (1.30, 1.85)***	1.57 (1.31, 1.87)***
Western	1.39 (1.16, 1.66)***	1.45 (1.21, 1.75)***	1.48 (1.22, 1.79)***
Nyanza	1.22 (1.02, 1.46)*	1.26 (1.05, 1.51)*	1.28 (1.06, 1.54)**
Nairobi	1.63 (1.33, 1.99)***	1.61 (1.31, 1.99)***	1.61 (1.31, 1.99)***
**Place of residence**			
Urban	1.00	1.00	1.00
Rural	0.83 (0.79, 0.89)***	0.89 (0.85, 0.94)***	0.89 (0.84, 0.94)***
**Employment status**			
Employed	1.00	1.00	1.00
Unemployed	0.85 (0.81, 0.89)***	0.88 (0.84, 0.93)***	0.93 (0.88, 0.99)*
**Household wealth quintiles**			
Richest		1.00	1.00
Richer		0.94 (0.88, 1.01)	0.95 (0.88, 1.02)
Middle		0.88 (0.81, 0.95)**	0.88 (0.82, 0.96)**
Poorer		0.89 (0.78, 0.93)**	0.86 (0.79, 0.94)**
Poorest		0.70 (0.64, 0.78)***	0.72 (0.65, 0.79)***
**Health insurance**			
Yes		1.00	1.00
No		0.73 (0.69, 0.78)***	0.74 (0.69, 0.79)***
**Age of respondents**			
45–49			1.00
40–44			1.04 (0.94, 1.15)
35–39			1.03 (0.93, 1.13)
30–34			1.02 (0.93, 1.13)
25–29			1.00 (0.91, 1.10)
20–24			0.94 (0.85, 1.03)
15–19			0.74 (0.65, 0.84)***
Log pseudo-likelihood	− 4629.853	− 4545.343	− 4522.237

***p < 0.001, **p < 0.01, *p < 0.05; OR for odds ratio; 95% CIs = 95% confidence intervals

## Discussion

Although there have been efforts to increase the screening rate of breast cancer in Kenya, little is known about factors associated with women’s breast cancer screening utilization. Using the Kenya Demographic and Health Survey and framing within the Andersen’s behavioural model of health care utilization, we contribute to the literature by examining whether predisposing, enabling, and need characteristics are correlated with women’s breast cancer screening utilization in Kenya.

We found that women with higher levels of education were more likely to have been screened for breast cancer than those with lower levels of education. This finding is consistent with previous studies that indicate that education is an important predictor of women’s utilization of healthcare services such as maternal health care in many African countries including Kenya [9]. As argued by Banchani and Tenkorang [27], education may provide an avenue for learning about health issues such as cancer screening for early detection and establishing awareness that healthcare utilization is important. Considering that very few women are knowledgeable about breast cancer in Kenya [23], women’s education may be critical for promoting breast cancer screening.

Our findings also indicate that never married women were less likely to have been screened for breast cancer than currently married women. This finding falls in line with the argument that marriage is associated with health benefits in terms of longevity, positive lifestyle choices, and healthcare utilization [31, 32]. Moreover, previous studies have revealed in Kenya that married women are more prone to using maternal health care services than unmarried women [9, 13]. Consistent with previous studies [10, 11], married people may be encouraged to make healthy lifestyle choices including access to health care services than unmarried people, due to emotional support and close monitoring from their spouses. Although the relationship between marriage and cancer screening has been widely established in developed countries [12, 33], this study may confirm that the benefit of marriage can be extended to breast cancer screening in developing countries such as Kenya.

Moreover, we found that women from the North Eastern Province were less likely to have been screened for breast cancer than women from any other province. This finding is similar to previous research, suggesting that adequate access to maternal care is particularly lacking in the North Eastern Province [34]. We argue that several characteristics of this province may serve as barriers to healthcare utilization among women. Specifically, the North Eastern Province is largely dominated by Muslims and Somalis. Remien et al. [31] find that Muslim women are often required to be accompanied by male partners or relative to visit healthcare facilities. Similarly, it is shown that access to health care services can sometimes be compromised among Muslim women, due to a religious obligation to avoid bodily exposure [35]. Coupled with these religious barriers, it is also possible that Somalis have limited opportunities to visit healthcare facilities, due to their nomadic nature [16]. As they are often constantly on the move and live far from urban areas where healthcare facilities may be available, geographical barriers to healthcare access may persist for Somali people [17]. These barriers may explain why women from the North Eastern Province have particularly low rates of breast cancer screening.

In accordance with previous research [20], we also found that women in rural areas were less likely to have been screened for breast cancer than those in urban areas. This finding is not surprising, as in the context of most developing countries including Kenya, rural communities often lack social infrastructure including hospitals and other health facilities [36]. There are challenges such as staffing shortages, lack of trained staff, insufficient space, and supply issues, which have been mentioned as determinants of inadequate service delivery in rural Kenya [37]. Given this skewed siting of health infrastructure, rural women may face an additional financial barrier, as they have to bear extra cost travelling to hospitals in urban areas to access cancer screening facilities [19]. Furthermore, it has been well established that rural women in Kenya are less likely to utilize health care services such as maternal health care and delivery in health facilities than urban women [9, 38]. Our study shows that women’s breast cancer screening utilization may follow this trend in Kenya.

In addition to lack of medical infrastructure in rural settings, it has also been widely argued that the cost of care is a major barrier to utilization of health care services in Kenya [37]. Research suggests that the cost for breast cancer screening is particularly high in Kenya. In the absence of universal health coverage to address issues of cost, only relatively wealthier women have access to health facilities for cancer screening [25]. Consistent with this argument, we found that poorer and unemployed women were less likely to have been screened for breast cancer than their richer and employed counterparts. Due to financial restrains, poor and unemployed women may not prioritize healthcare service utilization including breast cancer screening over other daily necessities such as food and housing [13, 39]. Moreover, Kenya’s national health insurance covers a substantial portion of costs in governmental hospitals [27]. Given our findings that uninsured women were less likely to have access to breast cancer screening than insured women, it is possible that health insurance creates an opportunity for women to utilize preventive health care services, regardless of women’s financial standing.

Furthermore, our findings show that older women were more likely to have been screened for breast cancer than younger women. This finding concurs with previous research, showing that age is positively associated with breast cancer screening in Namibia [8]. There are at least two reasons to explain these findings. First, older women may be more knowledgeable about health issues in general than younger women [40]. Second, breast cancer is age-specific in nature, putting older women at greater risk than younger women [1]. Reflecting on these conditions, it is possible that older women perceive more need to undergo breast cancer screening than younger women.

## Conclusion

Based on these findings, we have several policy recommendations. First, it may be important to increase women’s socioeconomic positions in Kenya. In particular, governmental and non-governmental initiatives should continue creating educational and employment opportunities for women. Moreover, there is need to provide affordable healthcare facilities for breast cancer screening, especially in rural communities. Based on the regional variations in breast cancer screening in Kenya, it may also be important to provide culturally and religiously sensitive breast cancer screening in the North Eastern Province. Also, it is critical to expand Kenya’s national health insurance, which can be useful in increasing the uptake of breast cancer screening among women with limited financial resources. Finally, there is an urgent need to intensify breast cancer awareness targeted at younger women in Kenya.

Despite these policy recommendations, there are some limitations to this study. For example, our findings are limited to statistical associations, as the Kenya Demographic and Health Survey is a cross-sectional survey. Moreover, although we used age of respondents as a proxy for need characteristics, it is also important to include attitudinal variables such as perceived risk levels towards breast cancer in future research [28]. Furthermore, given that breast cancer screening is culturally and religiously sensitive, responses may be influenced by social desirability bias. In addition, our source of data, the KDHS did not capture the timing to breast cancer screening which is considered essential in the early diagnoses and treatment of pre-cancerous lesions. Finally, our data was limited to women of reproductive ages (i.e. 15–49 years) although the risk of breast cancer includes women older than 49 years. To address these limitations, future research should employ longitudinal and qualitative approaches, which are useful for understanding the underlying processes that may shape women’s decision to utilize breast cancer screening. It will also be necessary for future studies in Kenya to collect information on timing to breast cancer screening. Survey instruments should be designed to capture the specific type of breast cancer screening (i.e. CBE or mammography).

## Data Availability

The data is publicly available on the website of the Demographic and Health Survey: https://dhsprogram.com/data/dataset/Kenya_Standard-DHS_2014.cfm?flag=1

## Abbreviations

KDHS: Kenya Demographic and Health Survey
KEMRI: Kenya Medical Research Institute
KNBS: Kenya National Bureau of Statistics
NACC: National AIDS Control Council
